# Virtual Screening of Protein Data Bank via Docking Simulation Identified the Role of Integrins in Growth Factor Signaling, the Allosteric Activation of Integrins, and P-Selectin as a New Integrin Ligand

**DOI:** 10.3390/cells12182265

**Published:** 2023-09-13

**Authors:** Yoshikazu Takada, Masaaki Fujita, Yoko K. Takada

**Affiliations:** 1 Department of Dermatology, UC Davis School of Medicine, Sacramento, CA 95817, USA; fujita.masaaki@e3.kepco.co.jp (M.F.); yoktakada@ucdavis.edu (Y.K.T.); 2 Department of Biochemistry and Molecular Medicine, UC Davis School of Medicine, Sacramento, CA 95817, USA

**Keywords:** integrins, receptors, growth factor signaling

## Abstract

Integrins were originally identified as receptors for extracellular matrix (ECM) and cell-surface molecules (e.g., VCAM-1 and ICAM-1). Later, we discovered that many soluble growth factors/cytokines bind to integrins and play a critical role in growth factor/cytokine signaling (growth factor–integrin crosstalk). We performed a virtual screening of protein data bank (PDB) using docking simulations with the integrin headpiece as a target. We showed that several growth factors (e.g., FGF1 and IGF1) induce a integrin-growth factor-cognate receptor ternary complex on the surface. Growth factor/cytokine mutants defective in integrin binding were defective in signaling functions and act as antagonists of growth factor signaling. Unexpectedly, several growth factor/cytokines activated integrins by binding to the allosteric site (site 2) in the integrin headpiece, which is distinct from the classical ligand (RGD)-binding site (site 1). Since 25-hydroxycholesterol, a major inflammatory mediator, binds to site 2, activates integrins, and induces inflammatory signaling (e.g., IL-6 and TNFα secretion), it has been proposed that site 2 is involved in inflammatory signaling. We showed that several inflammatory factors (CX3CL1, CXCL12, CCL5, sPLA2-IIA, and P-selectin) bind to site 2 and activate integrins. We propose that site 2 is involved in the pro-inflammatory action of these proteins and a potential therapeutic target. It has been well-established that platelet integrin αIIbβ3 is activated by signals from the inside of platelets induced by platelet agonists (inside-out signaling). In addition to the canonical inside-out signaling, we showed that αIIbβ3 can be allosterically activated by inflammatory cytokines/chemokines that are stored in platelet granules (e.g., CCL5, CXCL12) in the absence of inside-out signaling (e.g., soluble integrins in cell-free conditions). Thus, the allosteric activation may be involved in αIIbβ3 activation, platelet aggregation, and thrombosis. Inhibitory chemokine PF4 (CXCL4) binds to site 2 but did not activate integrins, Unexpectedly, we found that PF4/anti-PF4 complex was able to activate integrins, indicating that the anti-PF4 antibody changed the phenotype of PF4 from inhibitory to inflammatory. Since autoantibodies to PF4 are detected in vaccine-induced thrombocytopenic thrombosis (VIPP) and autoimmune diseases (e.g., SLE, and rheumatoid arthritis), we propose that this phenomenon is related to the pathogenesis of these diseases. P-selectin is known to bind exclusively to glycans (e.g., sLex) and involved in cell–cell interaction by binding to PSGL-1 (CD62P glycoprotein ligand-1). Unexpectedly, through docking simulation, we discovered that the P-selectin C-type lectin domain functions as an integrin ligand. It is interesting that no one has studied whether P-selectin binds to integrins in the last few decades. The integrin-binding site and glycan-binding site were close but distinct. Also, P-selectin lectin domain bound to site 2 and allosterically activated integrins.

## 1. Several Growth Factors Bind to the Classical Ligand-Binding Site (Site 1) of Integrins

**Figure 1:**
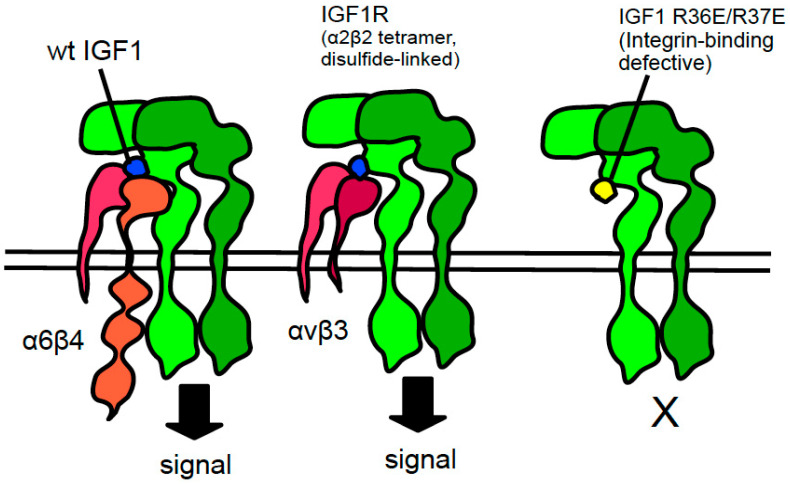
Integrin–IGF1–IGF1R ternary complex model. Type 1 insulin-like growth factor receptor (IGF1R) is a receptor tyrosine kinase that regulates cell growth and proliferation and can be activated by IGF1, IGF2, and insulin. IGF1R is proteolytically cleaved into the extracellular α chain and intracellular β chain (disulfide-linked). Two α and two β chains make up the IGF1R (α2β2). IGF1 binds to integrins αvβ3 and α6β4. This wild type IGF1 induces the integrin–IGF1–IGF1R ternary complex on the cell surface. This process is required for IGF1 signaling. The IGF1 mutant (R36E/R37E) is defective in integrin binding and ternary complex formation, although it still binds to IGF1R. R36E/R37E is defective in signaling and acts as a dominant-negative antagonist of IGF1 signaling and suppress tumor growth [10]. This indicates that IGF1 binding to IGF1R may not be sufficient for IGF1 signaling. This model can be applied to other growth factors, such as FGF1 [11], FGF2 [5], IGF2 [12], neuregulin-1 [7], and CX3CL1 [13].

Integrins are superfamily of cell surface heterodimers that recognize extracellular matrix (ECM), cell surface ligands (e.g., VCAM-1 and ICAM-1), and growth factors/cytokines. Integrins play important roles in normal biological processes (e.g., wound-healing and hemostasis) and in the pathogenesis of diseases [1,2]. Integrin–ligand interactions are thus a major therapeutic target. Through the virtual screening of a protein data bank (PDB) using docking simulation, we found that several growth factors (e.g., FGF1, FGF2, IGF1, IGF2, and neuregulin-1) [3,4,5,6,7] were bound to the classical ligand-binding site (site 1) of integrins, which was defined by the crystal structure of the RGD-αvβ3 complex [8,9]. These growth factors induced an integrin growth factor-cognate receptor ternary complex on the surface (ternary complex model) (Figure 1). Notably, the FGF1 mutant defective in integrin binding (Arg50 to Glu, R50E) was defective in signaling and ternary complex formation and suppressed tumor growth and angiogenesis (dominant-negative antagonists), although they still bind to FGF receptor [10,11]. We obtained similar results in FGF2, IGF1, IGF2, and neuregulin-1. We propose that the strategy we used is a useful platform for drug discovery.

The advantages of docking simulation is that we did not need any pre-knowledge or bias about potential integrin binding, and new integrin binding proteins are identified only based on docking energy. In most cases, we were able to prove that newly identified integrin ligands really bind to integrins as predicted by the simulation, and mutagenesis studies prove that the docking poses are correct. Most importantly, the newly found ligand binding to integrins have been shown to have important biological roles, as we describe in this review. The disadvantage of our strategy is that models of new ligands binding to integrins are so unexpected and often are not consistent with currently popular models (e.g., growth factor signaling and P-selectin). We are confident that the new integrin–ligand interaction model will eventually be generally accepted. Currently, we are trying to further develop potential therapeutics based on the antagonists we discovered, in addition to documenting as many new integrin ligands as possible.

## 2. Are Integrins Still Practical Targets for Anti-Cancer Therapy?

It has been shown that clinical trials with anti-integrin inhibitors have globally failed to demonstrate therapeutic benefits, and no inhibitors have been registered as anti-cancer drugs [14]. Our models of growth factor–integrin crosstalk suggest that growth factors binding to integrins and the resulting integrin–growth factor cognate receptor complex is required for growth factor signaling. If growth factors bind to multiple integrins, blocking one or two integrins may not be sufficient to block signaling. The blocking of one or two integrins may be compensated by others that bind to the same growth factors. We have shown that growth factor mutants defective in integrin binding (e.g., FGF1 R50E and IGF1 R36E/K37E) [10,11] result in the suppression of signaling and the mutants act as dominant-negative antagonists although the mutants still bind to cognate receptors (e.g., FGFR and IGF1R). Such mutations are expected to simultaneously block binding to multiple integrins. We propose that such dominant-negative mutants should be considered as potential therapeutics.

## 3. Several Chemokines Are Integrin Ligands

**Figure 2:**
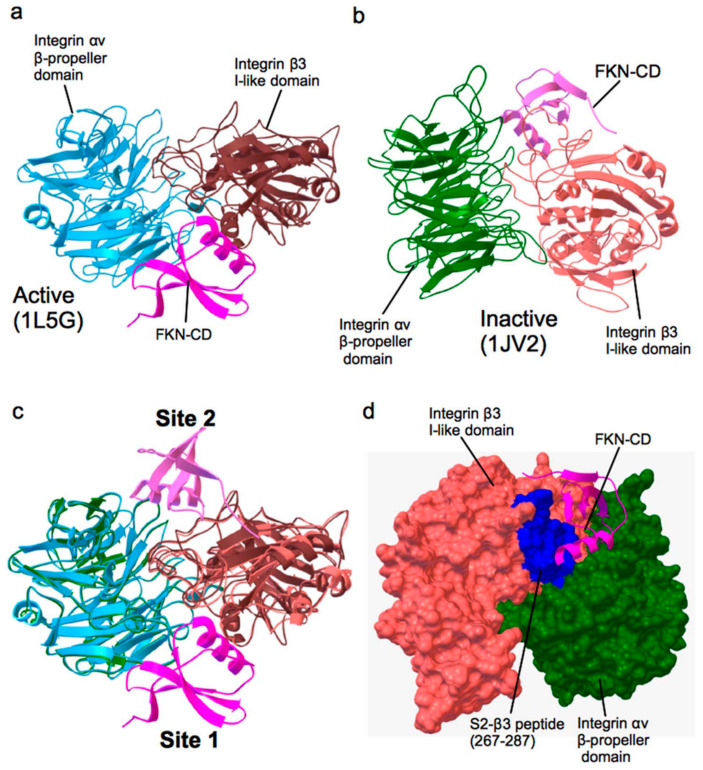
Docking simulation of CX3CL1 chemokine domain (FKN-CD) binding to αvβ3 with an inactive conformation predicting a new ligand-binding site (site 2). (**a**) A docking model of a FKN-CD–integrin αvβ3 (active) interaction [24]. The headpiece of ligand-bound form of integrin αvβ3 (PDB code 1L5G) was used as a target. The model predicts that FKN-CD (PDB code 1F2L, red) binds to the classical RGD-binding site of the integrin αvβ3 headpiece (site 1). (**b**) A docking model of FKN-CD–integrin αvβ3 (inactive) interaction. The headpiece of an inactive form of integrin αvβ3 (PDB code 1JV2) was used as a target. The model predicts the position of the second FKN-CD-binding site (site 2). (**c**) Superposition of two models shows that the positions of two predicted FKN-CD binding sites are distinct. (**d**) Position of the β3 peptide (267–287, blue) at site 2 (S2-β3). Most of amino acid residues in this peptide are predicted to interact with FKN-CD.

Unlike most chemokines, CX3CL1 is not synthesized by leukocytes but is expressed on the cell surface of IL-1- and TNFα-activated endothelium as a membrane-bound form [15]. Soluble CX3CL1 is released using metalloproteinases ADAM10 and ADAM17 [16,17,18]. CX3CL1′s highly selective receptor CX3CR1 (GPCR) is expressed in monocytes, T cells, NK cells, and neurons [19,20,21]. Interactions between membrane-bound CX3CL1 and CX3CR1 promote leukocyte adhesion to the endothelium [19,22,23]. We identified the chemokine domain of CX3CL1 (Fractalkine) [13] as a new ligand for integrins αvβ3, α4β1, and α5β1 and that CX3CL1 and integrins bind to CX3CR1 simultaneously [13] (Figure 2). The CX3CL1 mutant defective in integrin binding (K36E/R37E) was generated by introducing mutations in the predicted integrin-binding interface. This mutant was defective in signaling and suppressed CX3CL1 signaling as an antagonist, although it still bound to CX3CR1 [13], indicating that the direct integrin binding to CX3CL1 and subsequent integrin-CX3CL1-CXCR4 ternary complex formation is required for its signaling functions. The K36E/R37E mutant suppressed leukocyte recruitment in the peritonitis model, indicating that this mutant has therapeutic potential [13].

## 4. Several Growth Factors Bind to the Allosteric Site (Site 2) and Allosterically Activate Integrins

We unexpectedly discovered that CX3CL1 (fractalkine, FKN) activated soluble integrin αvβ3 in 1 mM Ca^2+^ in cell-free conditions [25] (Figure 2), indicating that this activation does not require inside-out signaling. Docking simulation between CX3CL1 chemokine domain (FKN-CD in Figure 2) and closed headpiece/inactive integrin αvβ3 (1JV2.pdb) predicted that CX3CL1 binds to a binding site that is distinct from the classical binding site (site 1). We realized that this new binding site should be an allosteric site (site 2) in an analogy to allosteric enzymes. So, we hypothesized that CX3CL1 can allosterically activate integrins. We discovered that CX3CL1 activated soluble integrin αvβ3 in 1 mM Ca^2+^ (to keep αvβ3 in an inactive state) in cell-free conditions in ELISA-type activation assays, as predicted from the docking simulation.

To study whether CX3CL1 activates αvβ3, the wells of 96-well microtiter plate were coated with a fibrinogen fragment (γC399tr), a specific ligand for αvβ3, and were blocked with BSA. Then, soluble αvβ3 ectodomain was incubated with immobilized γC399tr in the presence or absence of CX3CL1 at room temperature in 1 mM Ca^2+^. In the absence of CX3CL1, αvβ3 does not bind to γC399tr. We found that soluble αvβ3 bound to γC399tr in the presence of CX3CL1, indicating that CX3CL1 activated soluble αvβ3 in 1 mM Ca^2+^ in cell-free conditions.

This activation seems to require the direct binding of CX3CL1 to αvβ3, since the CX3CL1 mutant defective in integrin binding did not activate αvβ3. We assumed that this activation of soluble integrin should be allosteric and the second binding site should regulate the classical binding site, since otherwise CX3CL1 and the fibrinogen fragment, a specific ligand to αvβ3, will compete for binding to αvβ3, instead of facilitating binding. We identified the second ligand-binding site (site 2), which is distinct from the classical ligand-binding site (site 1) in the integrin headpiece via docking simulation using inactive/close headed integrin αvβ3 as a target. We showed that peptides from site 2 of the integrin β subunit bound to CX3CL1 and blocked integrin activation by CX3CL1 [25]. Therefore, we concluded that site 2 is the allosteric binding site and is involved in allosteric activation of integrins.

We showed that other integrin ligands secreted phospholipase A2 type IIA (sPLA2-IIA) [26], CXCL12 [27], and CD40L [28] activated integrins by binding to site 2, indicating that this mechanism of integrin activation is not limited to CX3CL1. sPLA2-IIA was previously shown to bind to site 1 [29].

It has been reported that 25-hydroxycholesterol, a lipid pro-inflammatory mediator, activates integrins by binding to site 2 of integrins and induced pro-inflammatory signals (e.g., NF-kB activation) [30]. It is likely that the ligand binding to site 2 induces pro-inflammatory signals.

## 5. Several Inflammatory Cytokines (CXCL12, CCL5, sPLA2-IIA, CD40L) Bind to and Activate Integrin αIIbβ3

Integrin αIIbβ3 is a receptor for several proteins, including fibronectin, fibrinogen, plasminogen, prothrombin, thrombospondin, vitronectin, and the von Willebrand factor (VWF). The activation of αIIbβ3 is a key event that triggers platelet aggregation by inducing αIIbβ3 binding to fibrinogen, leading to bridge formation between platelets [31,32]. It has been proposed that the activation of integrin αIIbβ3 is induced exclusively by the inside-out signaling of platelet agonists (e.g., thrombin, ADP, collagen), which bind to receptors on the cell surface. Signals received by other receptors induce the binding of talin and kindlin to the cytoplasmic end of the integrin β subunit at sites of actin polymerization [31,32,33]. It has, however, been proposed that canonical integrin activation pathways via platelet agonists induce integrin binding to multivalent ligands (e.g., ligand-mimetic antibody, Pac-1 IgM specific for αIIbβ3, with potential 10-binding sites) but do not enhance ligand binding affinity to monovalent ligands [34,35]. Since the activation of integrins through the binding of chemokines to site 2 (allosteric activation) enhanced the binding of monovalent ligands to integrins, we propose that site 2-mediated allosteric integrin activation is distinct from the integrin activation via the canonical inside-out signaling pathway.

## 6. Allosteric Activation by Chemokine Binding to Site 2

We wondered whether these chemokines bind to αIIbβ3 and activate this integrin in an allosteric manner. What is the contribution of inside-out signaling and allosteric activation? CXCL12 and CCL5 are stored in platelet granules and rapidly transported to the surface upon platelet activation [36], and CX3CL1 is expressed on activated endothelial cells. These chemokines are overproduced during inflammation (e.g., cytokine storm). It is unclear whether these chemokines play a role in the activation of αIIbβ3 on platelet activation or during inflammation. Here, we demonstrate that these chemokines are new ligands for αIIbβ3 and activated soluble and cell-surface αIIbβ3 by binding to site 2. The activation of αIIbβ3 by the chemokines was suppressed by peptides from site 2, indicating that their binding to site 2 is critical. They activated cell surface αIIbβ3 on CHO cells more quickly (half-maximal response within 1 min) and at much lower concentrations (1–10 ng/mL) of chemokines than in soluble αIIbβ3, probably because chemokines are concentrated by binding to cell surface proteoglycans. CHO cells do not have the machinery for inside-out signaling or cognate receptors for these chemokines, indicating that inside-out signaling or receptors for these chemokines are not involved. Notably, the activation of αIIbβ3 by the chemokines was much stronger than that of 1 mM Mn^2+^. These findings suggest that platelet integrin αIIbβ3 can be rapidly activated by pro-inflammatory chemokines independent of inside-out signaling. αIIbβ3 activation by pro-inflammatory chemokines is biologically relevant and possibly constitutes a missing link between inflammation and thrombosis.

The mechanism of integrin activation has been extensively studied in integrin αIIbβ3 as a model. αIIbβ3 is present as an inactive form and rapidly activated upon platelet activation. The activation of αIIbβ3 is a key event that triggers platelet aggregation by inducing αIIbβ3 binding to fibrinogen, leading to bridge formation between platelets [31,32]. It has been proposed that the activation of integrin αIIbβ3 is induced exclusively by inside-out signaling by platelet agonists (e.g., thrombin, ADP, collagen), which bind to receptors on the cell surface. Signals received by other receptors (e.g., the Thrombin receptor) induce the binding of talin and kindlin to the cytoplasmic end of the integrin β subunit at the sites of actin polymerization [31,32,33]. It has, however, been proposed that canonical integrin activation pathways by platelet agonists induce integrin binding to multivalent ligands (e.g., the ligand-mimetic antibody, Pac-1 IgM specific for αIIbβ3, with 10 potential binding sites) but do not enhance ligand-binding affinity to monovalent ligands [34,35].

Thus, we studied whether the platelet integrin αIIbβ3 can be activated by these chemokines in an allosteric manner upon platelet activation. Integrin αIIbβ3 is a receptor for several proteins, including fibronectin, fibrinogen, plasminogen, prothrombin, thrombospondin, vitronectin, and the von Willebrand factor (VWF). Since the activation of integrins via the binding of chemokines to site 2 (allosteric activation) enhanced the binding of monovalent ligands to integrins, we propose that site 2-mediated allosteric integrin activation is distinct from the integrin activation by the canonical inside-out signaling pathway.

## 7. Allosteric Activation of αIIbβ3 by Pro-Inflammatory Proteins

Importantly, many pro-inflammatory proteins (e.g., P-selectin, CCL5, CXCL12, CD40L) are stored in platelet granules (not CX3CL1) and rapidly transported to the surface. We thus hypothesized that αIIbβ3 on platelets can be allosterically activated by pro-inflammatory proteins stored in platelet granules upon platelet activation by binding to site 2. We discovered that these proteins bind to and allosterically activate integrins, including αIIbβ3 (see below). Chemokines are over-expressed during inflammation (e.g., cytokine storm and autoimmune diseases) and chemokines from outside of platelets may also allosterically activate αIIbβ3. Also, the transmembrane chemokine CX3CL1 is expressed on the surface of endothelial cells activated by pro-inflammatory cytokines [15] but not in the platelet granules. We hypothesize that CX3CL1 allosterically activates platelet αIIbβ3 and facilitates platelet adhesion to the vascular wall during inflammation. CXCL12 (SDF-1) is ubiquitously expressed in many tissues and cell types. CXCL12 is a potent chemoattractant for leukocytes and is believed to regulate signaling events through two different GPCRs, CXCR4 and CXCR7, in leukocytes [37,38,39,40]. The binding of CXCL12 to CXCR4 induces trimeric G protein signaling [41,42]. CXCL12 activated integrins αvβ3, α4β1, and α5β1 by binding to site 2 [27], indicating that this mechanism of integrin activation is not limited to CX3CL1. CCL5 (Rantes) is a pro-inflammatory chemokine, recruiting leukocytes to the site of inflammation.

## 8. CD40L Is an Integrin Ligand

CD40L is a key immunomodulatory factor and a major therapeutic target. CD40 is a cell surface receptor that belongs to the tumor necrosis factor-R (TNF-R) family and was first identified and functionally characterized on B lymphocytes [43]. Its critical role in T cell-dependent humoral immune responses was demonstrated by patients with the hyper-IgM syndrome type 1 (HIGM1), in which switching IgM to IgG is defective due to mutations in CD40L.

CD40L is a type II protein ligand member of the tumor necrosis factor (TNF) superfamily that, via interaction with CD40, is a key immunomodulatory factor responsible for modulating nearly all aspects of the adaptive immune response. CD40L is expressed as a transmembrane form and released as a soluble form (sCD40L) through proteolytic cleavage. The CD40L/CD40 interaction is required for enhancing antigen presenting the functions of dendritic cells, macrophages, and B cells; the maturation of humoral responses; and the enhancement of effector T cell responses [44]. CD40L is a key player in chronic autoimmune inflammatory diseases, including systemic lupus erythematosus (SLE), diabetes, multiple sclerosis (MS), and chronic kidney disease [24,45]. Clinical trials using humanized or chimeric anti-CD40L monoclonal antibodies blocking CD40L/CD40 interactions were undertaken in the early 2000s. Unfortunately, progress was halted due to the incidence of thromboembolic events in clinical trials. It is thus necessary to seek alternative ways to block CD40/CD40L signaling.

It has been believed that trimeric CD40L is biologically active but monomeric CD40L is not. CD40L levels markedly increase in certain pathologic conditions (SLE and RA), but CD40L exists mainly in a monomeric form [46]. CD40L simultaneously binds to integrin α5β1 and CD40, but it is unclear whether integrins and CD40 work together. The contribution of integrins to CD40/CD40L signaling has been largely ignored [47,48], and there is a huge gap of knowledge in this area. It has been reported that CD40L stabilizes arterial thrombi through binding to integrin αIIbβ3 [49]. αIIbβ3 recognizes the KGD motif at the N-terminus of CD40L (residues 115–117 of CD40L).

## 9. The Integrin-Binding Site of CD40L Is in the Trimeric Interface [6]

**Figure 3:**
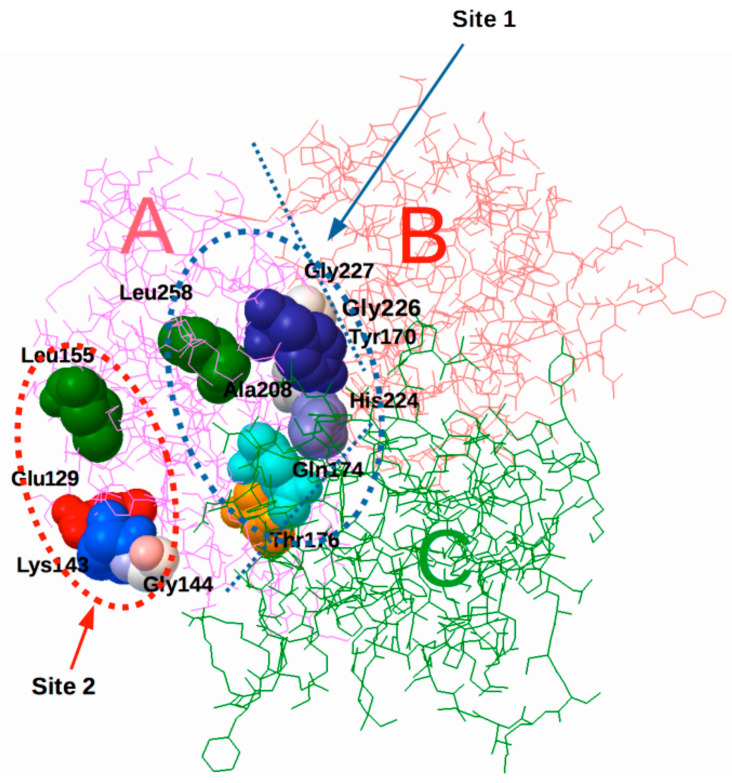
(**A**) The clustering of HIGM1 mutants at the site 1 and site 2 binding sites in CD40L. A previous study showed that eight HIGM1 mutants are clustered in the trimeric interface of CD40L (**A**–**C**) [6]. The four HIGM1 mutants are clustered at the site 2 binding site of CD40L on the outside of the CD40L trimer. In contrast to αvβ3, αIIbβ3 binding sites in CD40L are outside of the CD40L trimer [50].

We examined whether CD40L binds to integrin αvβ3 using a docking simulation between monomeric CD40L (PDB code 1ALY) and the headpiece of αvβ3 (PDB code 1L5G). The docking simulation predicts that monomeric CD40L will bind to the classical RGD-binding site of αvβ3 (site 1) (Figure 3). Notably, the integrin-binding site in CD40L is predicted to be in the trimeric interface.

Mutating amino acid residues at the predicted integrin-binding sites in CD40L reduces integrin binding. We found that mutating the amino acid residues in the predicted integrin-binding interface in the trimeric interface (Y170E, H224E/G226E, and G252E mutations) significantly reduced integrin binding. These findings suggest that the critical amino acid residues for integrin binding are in the trimeric interface of CD40L. These results are consistent with the docking model, indicate that integrins bind to monomeric sCD40L through binding sites that are cryptic in CD40L trimers.

## 10. The sCD40L Mutants Defective in Integrin Binding Still Bind to CD40

It is possible that the Y170E, H224E/G226E, and G252E mutations did not bind to integrin α5β1 because they were structurally not intact. We found, however, based on ELISA-type binding assays, that the sCD40L mutants defective in integrin binding still could bind to CD40. This indicates that they were properly folded and that the integrin-binding and CD40-binding sites are distinct.

The sCD40L mutants defective in integrin-binding are defective in inducing NF-kB activation. We found that wild type sCD40L induced robust NF-kB activation but sCD40L mutants defective in integrin binding did not induce NF-kB activation at all. These findings indicate that the sCD40L mutants defective in integrin binding are defective in signaling although they still bind to CD40, and that the binding of sCD40L to CD40 is not sufficient for signaling functions.

The sCD40L mutants defective in integrin binding are defective in inducing B cell activation. We studied the effect of sCD40L mutations on B cell activation. Enriched human B cells were cultured with or without soluble CD40L (wild type or 3 mutants), and their phenotype was analyzed using flow cytometry. We used the expression levels of CD80 as markers of B cell activation. Wild type sCD40L increased CD80 expression, whereas the rest of mutants did not. These findings indicate that the sCD40L mutants defective in integrin binding are defective in inducing B cell activation. It is thus highly likely that the binding of monomeric CD40L to integrins through the integrin-binding site in the trimeric interface is critically involved in CD40/CD40L activation via ternary complex formation.

## 11. Several HIGM1 Mutants Are Defective in Integrin Binding

Somatic mutations in CD40L result in HIGM1 [51]. The CD40L missense mutations induce defects in the structure and trimerization of CD40L, since the mutations are mostly located in the trimeric interface. Interestingly, we noticed that several HIGM1 mutations are clustered in the integrin-binding site in the trimeric interface of CD40L (Y170C, Q174R, T176I, A208D, H224Y, G226A, G227V). These HIGM1 mutants are not exposed to the surface in CD40L trimer. Six out of eight mutants were defective in integrin binding. Notably, these mutations had little or no effect on the binding of CD40. The HIGM1 mutants defective in integrin binding were defective in NF-kB activation using CD40L reporter cells. These findings indicate that several genetic mutations in the integrin-binding site within the trimeric interface reduced integrin binding and thereby induced CD40L functional defects, leading to HIGM1. These findings are consistent with the idea that direct integrin binding to monomeric CD40L plays a role in CD40/CD40L signaling. Therefore, we propose that the defective integrin binding may be a possible cause of defective CD40L functions in HIGM1. Integrin αvβ3 binds to CD40L in a KGD (residues 115–117 of CD40L)-independent manner.

Consistently, our docking simulation predicted that CD40L binds to αvβ3 with high affinity. We thus examined whether soluble integrin αvβ3 when fully activated by 1 mM Mn^2+^ binds to immobilized sCD40L using ELISA-type binding assays. We found that αvβ3 bound to the wild type sCD40L in a dose-dependent manner. These findings suggest that integrin αvβ3 binds to CD40L in the KGD-independent manner (since the soluble CD40L that we used does not have the KGD motif (residues 115–117) at the N-terminus). Mn^2+^ strongly supported the binding of soluble αvβ3 to immobilized sCD40L but Mg^2+^ and Ca^2+^ did not, indicating that αvβ3 should be fully activated. This is consistent with the idea that αvβ3 binds to CD40L. We found that the HIGM1 mutations (Y170C, Q174R, T176I, G227V, and L258S) reduced the binding of soluble αvβ3.

Also, several CD40L mutants defective in integrin binding and most HIGM1 mutants (which are also defective in integrin binding) were antagonists of CD40L/CD40 signaling. Thus, they have therapeutic potential.

## 12. CD40L Allosterically Activates Integrins by Binding to Site 2

Docking simulation predicted that CD40L binds to the allosteric site (site 2) as well as the classical ligand-binding site (site 1) of integrin αvβ3 [28]. We thus hypothesized that CD40L can allosterically activate integrins by binding to site 2. We found that CD40L bound to site 1 of soluble integrins αvβ3, α4β1, and α5β1 and activated them in an allosteric manner by binding to site 2 [28] (Figure 3). The binding interface of CD40L to these integrins is located on the outer surface of CD40L trimer, and another group of functionally defective HIGM1 mutants (S128R/E129G, L155P, K143T, and G144E mutants) were clustered in this region. We thus propose that CD40L binding to integrin site 1 and to CD40 and the subsequent integrin/CD40L/CD40 ternary complex formation is involved in CD40L/CD40 signaling and that integrin should be activated through the binding of CD40L to site 2 in advance.

## 13. CD40L Binds to Site 1 and Allosterically Activates Platelet Integrin αIIbβ3 by Binding to Site 2

CD40L is stored in platelet granules and rapidly transferred to the surface upon platelet activation by platelet agonists (e.g., thrombin and ADP). CD40L in activated platelets has been shown to play a role in atherosclerosis formation and stabilize the thrombus [52,53]. αIIbβ3 is known to recognize the KGD motif of CD40L (residues 115–117 of CD40L). α5β1 does not require the KGD motif [54]. However, the specifics of CD40L binding to αIIbβ3 are unclear. We showed that CD40L binds to αIIbβ3 in a KGD-independent manner using CD40L, which lacks the KGD motif. Two HIGM1 mutants, S128E/E129G and L155, reduced the binding of CD40L to the classical ligand-binding site (site 1) of αIIbβ3, indicating that αIIbβ3 binds to the outer surface of the CD40L trimer. Also, CD40L bound to site 2 of αIIbβ3 and allosterically activated αIIbβ3 without inside-out signaling. Two HIMG1 mutants, K143T and G144E, on the surface of trimeric CD40L suppressed CD40L-induced αIIbβ3 activation [50]. These findings suggest that CD40L binds to αIIbβ3 in a manner different from that of αvβ3 and α5β1 and induces αIIbβ3 activation. HIGM1 mutations are clustered in αIIbβ3 binding sites in CD40L and are predicted to suppress thrombus formation and immune responses through αIIbβ3.

## 14. P-Selectin (CD62P) C-Type Lectin Domain Functions as an Integrin Ligand

CD62P is a member of the selectin family and functions as a Ca^2+^-dependent receptor, which binds to the sialylated fucosylated sugars decorating receptors on neutrophils, monocytes, and platelets. CD62P is stored in the α-granules of platelets and Weibel–Palade bodies of endothelial cells [55] and is transferred to the plasma membrane upon activation [56,57]. The extracellular region of CD62P is composed of three different domains like other selectin types: a C-type lectin-like domain in the N-terminus, an EGF-like domain, and complement-binding protein-like domains with short consensus repeats (~60 amino acids). CD62P is anchored in the transmembrane region followed by a short cytoplasmic tail region [58]. CD62P mediates the rapid rolling of leukocytes over vascular surfaces during the initial steps in inflammation through interaction with CD62P glycoprotein ligand-1 (PSGL-1) [59]. Thus, it is a major therapeutic target for cardiovascular diseases, inflammation, and cancer metastasis [60]. Its recognition of the sialyl Lewis X via the lectin domain has exclusively been targeted for drug development, which highlights the opportunity to discover new drug targets to alternate ligands.

**Figure 4:**
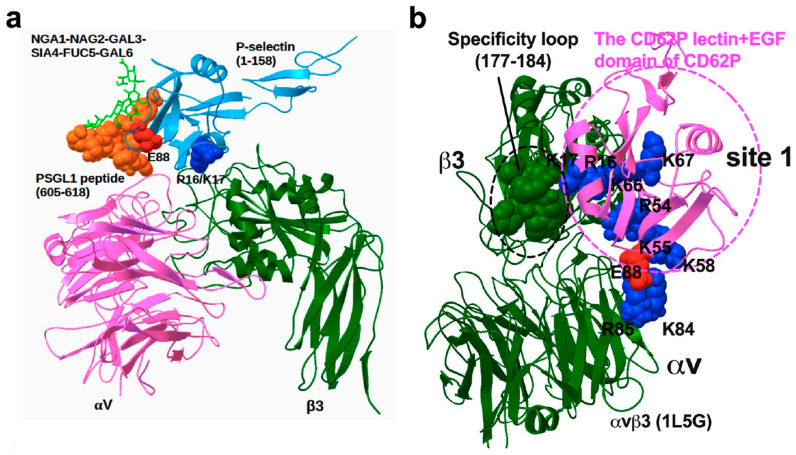
The CD62P lectin domain binding to integrin and PSGL-1. (**a**) The CD62P-αvβ3 docking model was superposed with the crystal structure of the CD62P–PSGL1 peptide complex (1G1S.pdb). The superposed model predicts that the integrin-binding site and PSGL-1-binding site are distinct. R16/K17 of the CD62P lectin domain is close to integrin αvβ3, and E88 of the lectin domain is close to PSGL-1 peptide and glycan. (**b**) Docking simulation of the interaction between open/active αvβ3(1L5G.pdb) and the CD62P lectin domain (1G1Q.pdb) was performed using Autodock3. The specificity loop (residues 177–184 of β3) is next to site 1. This loop is located between site 1 and site 2.

**Figure 5:**
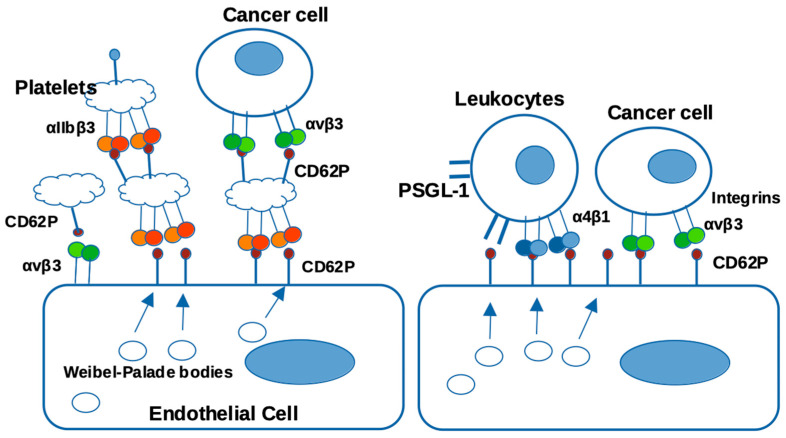
Potential role of CD62P binding to integrins. CD62P is stored in endothelial cells and platelet granules and transported to the surface upon endothelial or platelet activation. It has been proposed that CD62P plays a role in cell–cell interactions during leukocyte extravasation or cancer metastasis by binding to P-selectin glycan ligand-1 (PSGL-1) but the expression of PSGL-1 is limited to leukocytes. P-selectin–integrin interaction is expected to play a role in cell–cell interactions and signaling, since integrins are widely expressed.

The virtual screening of the protein data bank provided the basis of our CD62P-αvβ3 docking model, which predicts that the ligand-binding site of integrins resides within site 1 and is distinct from the glycan-binding site of CD62P [61] (Figure 4). We identified amino acid residues critical for integrin binding in the lectin domain by introducing mutations in the predicted integrin-binding site (e.g., the K16E/R17E mutation). The E88D mutation that is known to block glycan binding [62] minimally affected integrin binding. These findings suggest that CD62P acts as an integrin ligand on activated endothelial cells, leukocytes, and platelets. Further, it is possible that CD62P mediates cell–cell interactions by binding to integrins (Figure 5) simultaneously to its capacity to mediate glycan binding and rolling. In addition, the lectin domain of CD62P binding to site 2 can activate integrin and thus mediate outside-in signaling during platelet and leukocyte recruitment [61].

## 15. PF4 Is an Inhibitory Chemokine: Anti-PF4 Changes the Phenotype of Inhibitory Chemokine PF4 to Pro-Inflammatory Chemokine

As the novel SARS-CoV-2 virus continues to infect numerous individuals worldwide, one of the leading approaches in dealing with the global health crisis is vaccination against COVID-19. It has recently been reported that vaccination may result in a vaccine-induced catastrophic thrombotic thrombocytopenia (VITT) disorder. This disorder presents as extensive thrombosis in atypical sites, primarily in the cerebral venous, alongside thrombocytopenia and the production of autoantibody against platelet-factor 4 (PF4, chemokine CXCL4). This rare adverse effect extremely resembles the clinical presentation of the classical immune-mediated heparin-induced thrombocytopenia (HIT) disorder, which is induced by the anti-PF4/heparin complex and occurs following exposure to heparin. VITT is also very similar to autoimmune HIT (aHIT), which is induced by anti-PF4 but none of these patients had been pre-exposed to heparin before disease onset. Anti-PF4 autoantibodies have also been detected in several autoimmune diseases (e.g., SLE, systemic sclerosis, and RA) [63,64,65].

PF4 is one of the most abundant proteins in platelet granules and rapidly transported to the surface upon platelet activation. PF4 is present at >1 μg/mL concentrations in plasma. The activation of platelet integrin αIIbβ3 is a key event that leads to αIIbβ3 binding to fibrinogen and platelet aggregation. Current models of thrombotic thrombocytopenia (TT) do not include the activation of αIIbβ3. Chemokines CX3CL1 and CXCL12 are ligands for several integrins and activate integrins in an allosteric mechanism by binding to the allosteric site (site 2) of these integrins, which is distinct from the classical ligand-binding site (site 1). We suspected that PF4 binds to integrin αIIbβ3 and allosterically activates it. We showed that PF4 binds to soluble integrin αIIbβ3 in cell-free conditions but did not activate this integrin at physiological PF4 concentrations (<1 μg/mL). Notably, an anti-PF4 (RTO)/PF4 complex potently activated soluble αIIbβ3 in a heparin-independent manner [50]. We propose that anti-PF4 changed the conformation of PF4 and strongly activates αIIbβ3 by binding to site 2 and results in the strong aggregation of platelets. Since RTO does not require heparin, the PF4/anti-PF4-induced αIIbβ3 activation may represent aHIT or VITT but not HIT. Our studies connect anti-PF4, PF4, the activation of αIIbβ3, and subsequent platelet aggregation. We also showed that PF4/anti-PF4 potently activated vascular integrin αvβ3, which may play a critical role in autoimmune diseases. We have developed a PF4 mutant that does not induce αIIbβ3 and αvβ3 activation by mutating the site 2 binding site in PF4 (predicted by docking simulation). The PF4 mutant acted as an antagonist of the anti-PF4/PF4-induced activation of integrins in ELISA-type activation assays, indicating that it has potential as a therapeutic [50].

## 16. Conclusions

**Figure 6:**
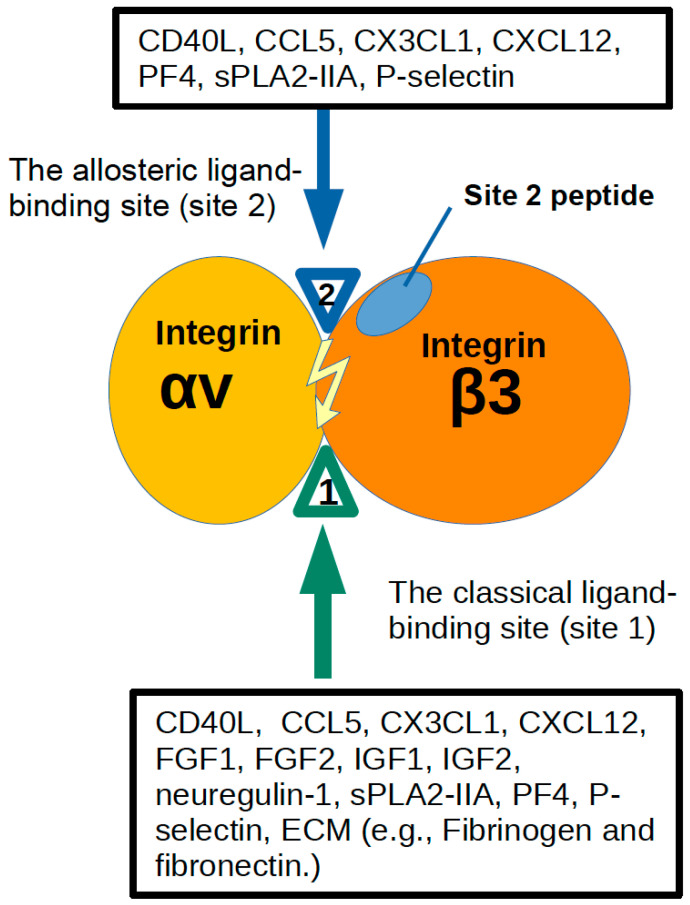
Ligand binding to site 1 and site 2 of αvβ3 and αIIbβ3. Docking simulation predicts that several growth factors/cytokines bind to the classical ligand (RGD) binding site (site 1) and the allosteric site (site 2) in the integrin headpiece. Site 2 is distinct from site 1 and is on the opposite side of site 1. Integrins are not activated in normal conditions. From analogy to allosteric enzymes, site 1 is not open but site 2 is open. It is expected that ligand binding to site 2 of inactive integrins will result in the opening of site 1 (allosteric activation). It appears that this process does not require canonical inside-out signaling (since these ligands activate soluble integrin ectodomain in cell-free conditions) and does not accompany global conformational changes. We propose that allosteric activation is distinct from integrin activation by well-established inside-out signaling. This model can be applied to αIIbβ3 as well. Notably, most of the allosteric activators that bind to site 2 (e.g., CD40L, CCL5, CXCL12, PF4, and P-selectin) are stored in platelet granules and are rapidly transported to the surface upon platelet activation. Allosteric activation may thus be involved in the activation of αIIbβ3 and may trigger platelet aggregation.

We identified many integrin ligands through the virtual screening of a protein data bank with the integrin headpiece as a target. Our studies showed that integrins are co-receptors for several growth factors that have different structures and bind to different receptors. Also, we showed that the direct binding to these growth factors to integrins to the classical ligand-binding site (site 1) is required for their signaling functions through the integrin–growth factor–growth factor receptor ternary complex on the cell surface. The growth factor mutants defective in integrin binding are defective in signaling and ternary complex formation, although the mutants still bind to growth factor receptors, indicating that growth factor binding to their cognate receptor is not sufficient for signaling functions. Furthermore, the growth factor mutants defective in integrin binding act as antagonists for signaling functions and suppressed signaling via wild type growth factors (dominant-negative effect). Integrins have an allosteric binding site (site 2), which is distinct from site 1, and many growth factors or pro-inflammatory factors bind to site 2 and activate integrins (opening of site 1) (Figure 6). An inflammatory mediator (25HC) is shown to bind to site 2, activates integrins and induce inflammatory signals, indicating that site 2 is involved in inflammatory signals. Notably, site 2-derived peptides bind to many growth factors/cytokines and blocked allosteric activation, indicating that site 2 peptides have therapeutic potential. The virtual screening of a protein data bank identified an unexpected integrin ligand P-selectin lectin domain. P-selectin binding to integrins and resulting P-selectin signaling and cell–cell interactions are expected to play a role in inflammation and cancer metastasis in addition to P-selectin–PSGL-1 interaction. The inhibitory chemokine PF4 (CXCL4) binds to site 2 but does not activate integrins, indicating that PF4 is a potential site 2 antagonist. Surprisingly, an anti-PF4 antibody changes the phenotype of PF4 from inhibitory to inflammatory. This may be related to the pathogenesis of autoimmune thrombosis or inflammatory diseases, and autoantibodies against PF4 have been detected to these diseases.

Biological and pathological roles of the allosteric activation of integrins. We discovered that the direct binding of growth factors/cytokines to integrins (site 1) and subsequent integrin–cytokine–cognate receptor ternary complex formation is critical for cytokine signaling. Integrins in normal conditions may not be activated in our body fluid with high Ca^2+^ (1 mM). We propose that allosteric activation may enhance cytokine signaling by facilitating ternary cytokine binding to integrins in pathological situations in which levels of inflammatory cytokines in body fluid are increased (e.g., cytokine storm). The activation of αIIbβ3 via allosteric activation is expected to trigger platelet aggregation. We discovered that the platelet integrin αIIbβ3 is allosterically activated through several inflammatory proteins (chemokines, CD40L, and CD62P), which are stored in platelet granules and transported to the surface upon platelet activation. Therefore, the allosteric activation of integrins by inflammatory proteins is a potential therapeutic target.
